# Week 96 Results of Bictegravir/Emtricitabine/Tenofovir Alafenamide for HIV Treatment in People With Substance Use Disorders

**DOI:** 10.1093/ofid/ofae737

**Published:** 2024-12-20

**Authors:** Joshua P Havens, Sara H Bares, Elizabeth Lyden, Nada Fadul, Susan Swindells

**Affiliations:** College of Pharmacy, University of Nebraska Medical Center, Omaha, Nebraska, USA; College of Medicine, University of Nebraska Medical Center, Omaha, Nebraska, USA; College of Medicine, University of Nebraska Medical Center, Omaha, Nebraska, USA; Department of Biostatistics, University of Nebraska Medical Center, Omaha, Nebraska, USA; College of Medicine, University of Nebraska Medical Center, Omaha, Nebraska, USA; College of Medicine, University of Nebraska Medical Center, Omaha, Nebraska, USA

**Keywords:** adherence, bictegravir/emtricitabine/tenofovir alafenamide, HIV, substance use disorders, viral suppression

## Abstract

**Background:**

The BASE study (NCT03998176), a phase 4, 48-week (W), single-arm, prospective trial, revealed that the use of bictegravir/emtricitabine/tenofovir alafenamide (B/F/TAF) in people with HIV and substance use disorders (PWH/SUD) was safe and effective without emergent antiretroviral resistance despite incomplete adherence. Here, we present the W96 results.

**Methods:**

A retrospective analysis of all participants enrolled in the BASE study was completed from W48 to W96. End points of interest at W96 included the proportion of participants with viral suppression (VS; HIV RNA <50 copies/mL [c/mL]), incidence of protocol-defined virologic failure (PDVF; 2 consecutive ≥400 c/mL), safety, adherence (percentage of days covered [PDC]), retention in care, and prevalence of ongoing substance use.

**Results:**

All enrolled BASE participants (n = 43) were included in the W96 analysis. At W48, 21 participants (49%) had achieved VS (intent-to-treat [ITT]). Thirty-six (84%) participants completed W96, with 19 achieving an HIV RNA <50 copies/mL (ITT, 44%; per-protocol, 54%). Seven participants (19%) met PDVF; genotyping was performed on 2, with no evidence of treatment-emergent antiretroviral resistance noted. No safety signals were identified or attributed to B/F/TAF. Adherence to B/F/TAF decreased 18% after W48 (mean PDC: W0–W48, 72%; W48–W96, 54%; *P* < .01). Participants exhibiting adherence rates of ≥4 doses/wk (PDC ≥57%) were more likely to achieve VS (PDC ≥57%, 84.2%, vs PDC <57%, 15.8%; *P* < .01). Retention in care remained stable, and participants continued to use substances through W96.

**Conclusions:**

At W96, the proportion of PWH/SUD achieving VS with B/F/TAF decreased to 44%, along with an adherence decrease of 18%, with no evidence of treatment-emergent HIV drug resistance occurring.

## Graphical Abstract

**Graphical Abstract:**
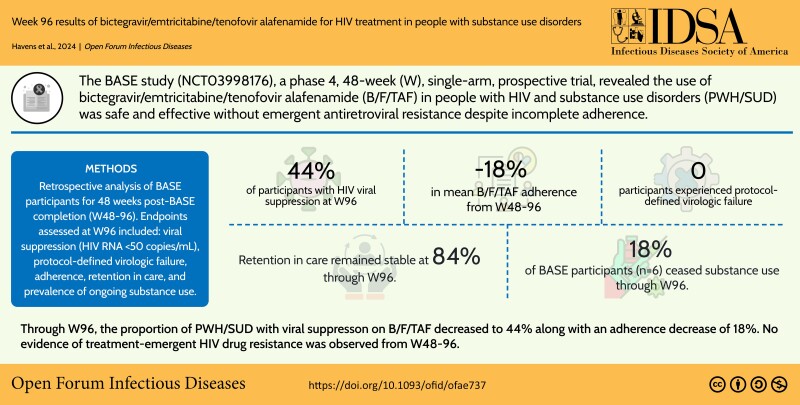
This graphical abstract is also available at Tidbit URL: https://tidbitapp.io/tidbits/week-96-results-of-bictegravir-emtricitabine-tenofovir-alafenamide-for-hiv-treatment-in-people-with-substance-use-disorders?utm_campaign=tidbitlinkshare&utm_source=IO

Substance use disorders (SUDs) among people with HIV (PWH) remain a growing concern in the United States, with an estimated prevalence rate of 48% and nearly 20% reporting polysubstance use [1]. Injection drug use (IDU) is an important driver of the HIV epidemic, with 7% of people with newly diagnosed HIV reporting IDU [2]. Success of HIV care among people with HIV and SUD (PWH/SUD) has been limited by lower rates of antiretroviral treatment (ART) adherence and suboptimal engagement in care [3–10], leading to poor treatment outcomes and increased HIV transmission [9, 11, 12]. As a result of these dynamics, a heightened focus on addressing SUD among PWH has ensued, notably within the Ending the HIV Epidemic initiative released in 2019 [13].

Bictegravir/emtricitabine/tenofovir alafenamide is a co-formulated, integrase strand transfer inhibitor (INSTI)–based ART regimen currently listed as a preferred treatment option for most PWH in the United States [14]. B/F/TAF exhibits a favorable safety profile, high rates of viral suppression (VS; 89%–96%), low drug–drug interaction risk, and a high barrier to antiretroviral resistance [15–19]. The BASE study (NCT03998176) was a phase 4, 48-week (W), open-label, single-arm, prospective trial evaluating the use of B/F/TAF in PWH/SUD [20]. Key findings included a decrease in VS through W48 (proportion with HIV RNA <50 copies/mL [c/mL]; intent-to-treat [ITT]: W24, 72%; W48, 49%) and a decline in retention to study visits. Despite waning VS, BASE participants exhibited rates of B/F/TAF adherence that were equivalent to 5–6 weekly doses of B/F/TAF as measured by tenofovir diphosphate dried blood spot concentrations. Further, decreasing use of substances, primarily methamphetamines, and improvements in quality of life (ie, Short Form-12 [SF-12] physical/mental scores) were reported over the course of the study. Development of antiretroviral resistance occurred at W48 in 1 participant with selection of an M184V; however, resuppression occurred with continuation of B/F/TAF [20].

Herein, we present the retrospective results of the W96 analysis of the BASE study. Our analysis reports the efficacy, safety, adherence, retention in care, and SUD characteristics of participants enrolled in the BASE study.

## METHODS

### Study Design and Participants

The BASE study (NCT03998176) was a phase 4, open-label, single-arm, prospective 48-week trial evaluating the use of B/F/TAF in PWH with ongoing SUD at the Specialty Care Center in Omaha (NE, USA). The study design and inclusion/exclusion criteria have been previously published [20]. Briefly, eligible participants included adults (≥19 years) who were ART treatment–naïve or –experienced and had an HIV-1 RNA ≥1000 c/mL without archived resistance to any of the components of B/F/TAF (presence of thymidine analog mutations [TAM] or the M184V/I mutation was allowed) or endorsement of any substance use within the past 6 months of enrollment excluding nicotine, alcohol, or marijuana.

BASE participants were initiated on B/F/TAF, followed through W48 (W0–W48, primary study period), and then returned to routine clinical care. Data for W48–W96 were collected retrospectively (W48–W96, retrospective study period). We identified clinical visits that occurred within +/− 8 weeks of 96 weeks from initial BASE study entry, designating this the W96 end point (herein, W96). Clinical outcomes occurring after the W96 period were not assessed. The ITT population included all enrolled BASE participants. Adherence to B/F/TAF was assessed by refill histories, which were utilized to calculate a percentage of days covered (PDC) through W96. Safety was assessed by laboratory tests during clinical care and B/F/TAF discontinuation, and reported adverse events (AEs) were graded according to the DAIDS Table for Grading the Severity of Adult and Pediatric Adverse Events [21]. Protocol-defined virologic failure (PDVF) was defined as HIV-1 RNA >400 c/mL on 2 consecutive readings at week 24 or later.

The University of Nebraska Medical Center Institutional Review Board (IRB) approved the protocol (IRB number 471-19-FB). All participants provided written informed consent for W0–W48. An independent safety monitoring committee provided study oversight during the primary study period. IRB approval was received for the retrospective study period through W96.

### End Points and Assessments

The BASE study primary analysis was the proportion of participants with HIV RNA <50 c/mL at week 24 by US Food and Drug Administration (FDA) Snapshot analysis for the ITT population, as previously published [20]. End points assessed at W96 included proportion of participants with HIV RNA <50 c/mL, incidence of PDVF, development of treatment-emergent antiretroviral resistance, proportion of participants with AEs, retention in care (proportion of participants completing W96 and number of missed visits), adherence (PDC), and incidence of SUD during W48–W96 (ie, reported in the electronic health record).

### Statistical Analysis

Descriptive statistics were used to summarize the data. Counts and percentages were used for categorical data, with median (range) or mean (SD) used for continuous data. HIV RNA was dichotomized at ≥ or <50 c/mL and ≥ or <200 c/mL. ITT (participants receiving ≥1 dose of B/F/TAF) and observed, per-protocol analyses were performed for VS end points through W96. The Fisher exact test was used to compare the proportion of participants achieving an HIV RNA <50 c/mL among adherence subgroups. Paired *t* tests were used to compare mean differences in PDC, weight, body mass index (BMI), and missed visit frequency between the primary study period (W0–W48) and the retrospective analysis period (W48–W96). All analyses were done using SAS, version 9.4 (SAS Institute Inc., Cary, NC, USA).

## RESULTS

### Study Population and Participant Disposition

BASE participant baseline characteristics have been previously published [20]. Briefly, the median (range) age was 38 (21–62) years; most were cisgender men (79.1%), White (81.4%), and Latinx (16.3%). Methamphetamine SUD was most common (95.3%). Most were treatment-experienced (n = 31, 72.1%). Roughly half (n = 18, 58.1%) had archived resistance-associated mutations (RAMs); non-nucleoside reverse transcriptase inhibitor (NNRTI) RAMs (28%) and M184 V/I mutations were most common (16.1%).

Patient disposition through W96 is outlined in Supplementary Figure 1. Thirty-one (72.1%) and 36 participants (83.7%) completed W48 and W96, respectively. Of the 31 participants who completed W48, 2 were lost to follow-up and did not complete W96. Seven participants who did not complete W48 reengaged in care during W48–W96.

### Effectiveness

**Figure 1.**
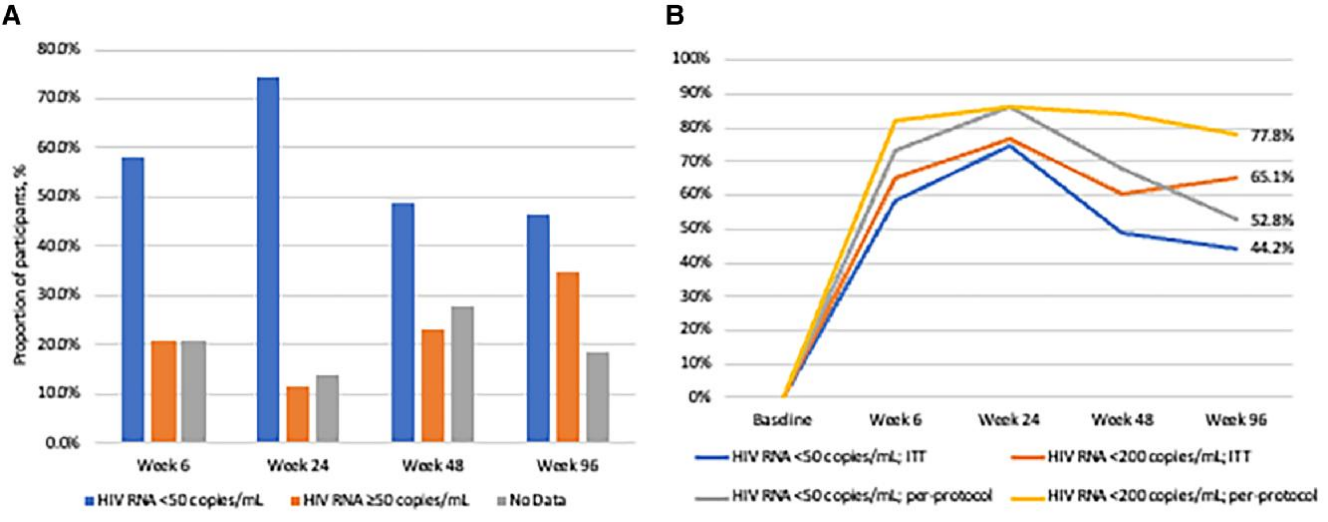
Week 96 virologic outcomes. *A*, FDA Snapshot algorithm by ITT analysis. *B*, Proportion of participants who were virologically suppressed by ITT and per-protocol analyses. Abbreviations: FDA, Food and Drug Administration; ITT, intent-to-treat.

At W96, 19 participants (44.2%) achieved an HIV RNA <50 c/mL (FDA Snapshot, ITT) (Figure 1*A*). Seven participants (16.3%) did not provide HIV RNA results or other clinical data for the W96 period. Of the 17 participants (39.5%) with HIV RNA ≥50 c/mL at W96, the median (range) HIV RNA was 130 (54–2 200 000) c/mL. Seven participants (n = 7/17, 41.2%) met the criteria for PDVF at W96 (Supplementary Table 1). Two participants returned for repeat HIV RNA and resistance testing (HIV-1 drug resistance by next-generation sequencing [22]) after W96. Disposition of the other 5 participants was: 1 was lost to follow-up after W96, 1 died shortly after W96 (motor vehicle accident), and 3 participants had reengaged in care at W96 with no antiretroviral doses taken in the prior 30 days (subjective reporting). No evidence of treatment-emergent resistance was observed from either participant with HIV resistance testing performed. One participant was transitioned to dolutegravir (DTG) and F/TAF due to nausea while on B/F/TAF. All other participants continued B/F/TAF. In the observed, per-protocol analyses, 52.8% (n = 19/36) achieved HIV-1 RNA <50 c/mL. Rates of VS by an HIV RNA threshold of <200 c/mL for the ITT and observed, per-protocol populations were 65.1% (n = 28/43) and 77.8% (n = 28/36), respectively (Figure 1*B*).

**Figure 2.**
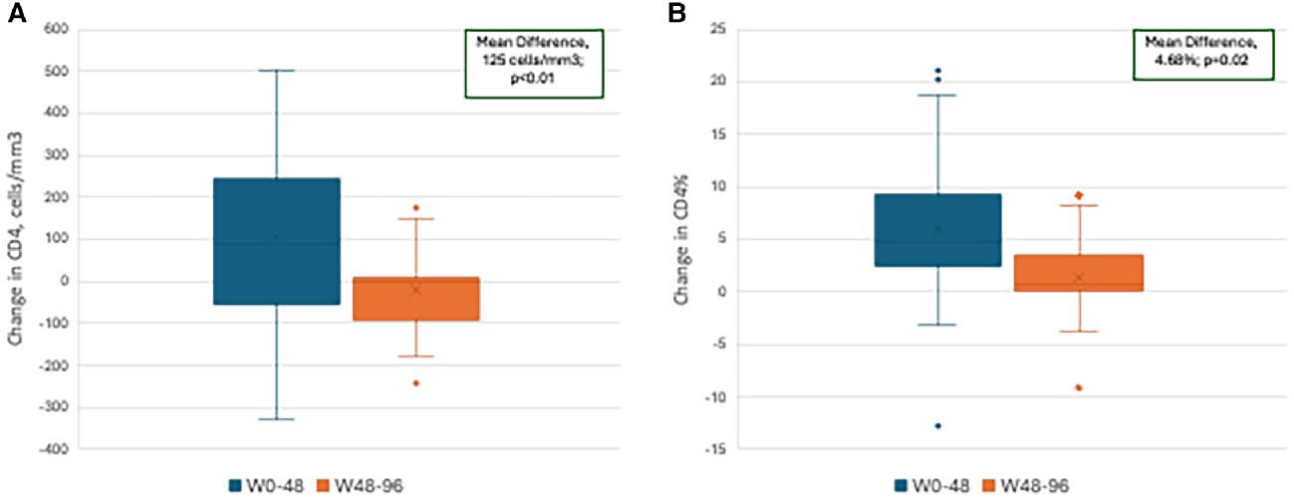
Mean change in CD4 and CD4% during the study (W0–W48) compared with poststudy (W48–W96). *A*, CD4. *B*, CD4%. Comparison for the observed population (W0–W48, 31 participants; W48–W96, 36 participants). Abbreviation: W, week.

Immunologic metrics at W96 revealed mean CD4 and CD4% of 551.7 cells/mm^3^ and 27.6%, respectively. From W0 to W96, notable increases in mean (SD) CD4 (82.7 [207.5] cells/mm^3^) and CD4% (5.3% [8.7%]) were observed. Significant differences were observed when comparing the mean changes in CD4 and CD4% of W0–W48 vs W48–W96 (CD4, W0–W48: 104.7 cells/mm^3^; vs W48–W96: −20.39 cells/mm^3^; *P* < .01; CD4%, W0–W48: 5.1%; vs W48–W96: 1.3%; *P* = .02) (Figure 2).

### Safety

Adverse events from W48 to W96 are summarized in Table 1. Overall, 23 participants (53.5%) reported 36 AEs, with none attributed to B/F/TAF. Most AEs were incident infections (n = 19, 48.7%), with 5 participants (11.6%) diagnosed with syphilis. Four neuropsychiatric events (11.1%) occurred among 3 participants. Notably, suicidal ideation occurred twice in 1 participant during periods of methamphetamine intoxication. Laboratory AEs were relatively uncommon and occurred on 4 occasions among 4 participants. There were no grade 4 severe AEs, and no AEs led to discontinuation of B/F/TAF.

**Table 1.** Summary Adverse Events, Week 48 Through Week 96

	B/F/TAF, Week 48–96	
…	Participants No. (%)^a^	Cumulative AEs No. (%)
Any AE	23 (53.5)	36 (100.0)
AE, grades 1–3	31 (72.1)	32 (88.9)
Infectious etiology	19 (44.2)	19 (48.7)
Syphilis	5 (11.6)	5 (13.9)
COVID-19	2 (4.7)	2 (5.6)
Bacteremia	2 (4.7)	2 (5.6)
Cellulitis	1 (2.3)	1 (2.8)
Pneumonia	1 (2.3)	1 (2.8)
Hepatitis C	1 (2.3)	1 (2.8)
Other^b^	7 (16.3)	7 (19.4)
Neuropsychiatric events	3 (7.0)	4 (11.1)
Suicidal ideation	1 (2.3)	2 (5.6)
Psychosis	1 (2.3)	1 (2.8)
Hallucinations	1 (2.3)	1 (2.8)
Abscess	3 (7.0)	3 (8.3)
Acute kidney injury	1 (2.3)	1 (2.8)
Atrial flutter	1 (2.3)	1 (2.8)
Tenosynovitis	1 (2.3)	1 (2.8)
Pulmonary embolism	1 (2.3)	1 (2.8)
Pyelonephritis	1 (2.3)	1 (2.8)
Stabbing	1 (2.3)	1 (2.8)
Grade 4	0 (0.0)	0 (0.0)
Elevated TSH, >10 µIU/mL	1 (2.3)	1 (2.8)
Proteinuria, 3+	1 (2.3)	1 (2.8)
Grade 4	0 (0.0)	0 (0.0)
Laboratory AE, grade 1–3	4 (7.0)	4 (11.1)
Hemoglobin A1c, >10%	1 (2.3)	1 (2.8)
Elevated PSA, >4 ng/mL	1 (2.3)	1 (2.8)
Drug-related AE	0 (0.0)	0 (0.0)

Abbreviations: AE, adverse event; B/F/TAF, bictegravir/emtricitabine/tenofovir alafenamide; COVID-19, coronavirus disease 2019; PSA, prostate-specific antigen; TSH, thyroid-stimulating hormone.

^a^All percentages were calculated based on the total number of participants in the intent-to-treat analysis, n = 43.

^b^Other infectious etiology included: *Giardia* spp., *Shigella* spp., *Clostridium difficile*, histoplasmosis, and MPox infections.

The mean (SD) weight and BMI change from W0 to W96 were 5.2 (9.0) kg and 1.5 (2.6)  kg/m^2^. Mean weight and BMI were similar during the primary study period (W0–W48) compared with the retrospective analysis period (W48–W96; weight, W0–W48: 2.8 kg; vs W48–W96: 2.3 kg; *P* = .72; BMI, W0–W48: 0.8 kg/m^2^; vs W48–W96: 0.7 kg/m^2^; *P* = .76) (Supplementary Figure 2A, B).

### Adherence

**Figure 3.**
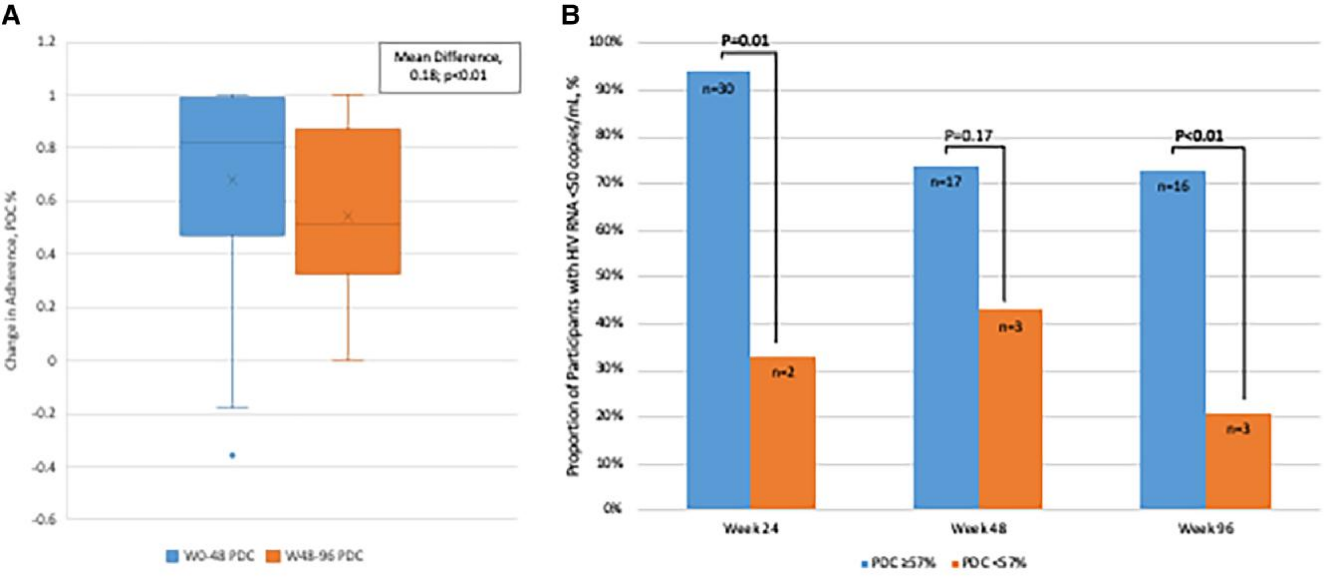
B/F/TAF adherence through week 96. *A*, Mean change in adherence by PDC (W0–W48) compared with poststudy (W48–W96). *B*, Proportion of participants with virologic suppression (HIV RNA <50 copies/mL) by B/F/TAF adherence threshold of ∼4 doses/wk (PDC). Abbreviations: B/F/TAF, bictegravir/emtricitabine/tenofovir alafenamide; PDC, percentage of days covered; W, week.

Through W96, the mean (SD) adherence by PDC was 62.1% (25.7%). During the retrospective study period (W48–W96), adherence was 54.4%, representing an 18% decrease compared with the primary study period (W0–W48, 72.5%; *P* < .01) (Figure 3*A*). Similarly, significantly higher rates of adherence were observed among participants achieving VS compared with those with HIV viremia at W96 (VS, 78.0%; vs viremic, 46.1%; *P* < .01) (Table 2). Virologic status changes from W48 to W96 occurred in 13 participants (30.2%), with 6 participants (16.7%) achieving VS (median [range] HIV RNA, 0 [0–0]) and 7 participants (19.4%) losing VS at W96 (median [range] HIV RNA, 93 [54–1190]) (Table 2). Compared with W0–W48, adherence changed +3.0% and −55.0% from W48 to W96 for the participants achieving VS and losing VS, respectively. Ten participants (27.8%) remained viremic from W48 to W96 (median [range] HIV RNA, 1315 [67–2 200 000]), with a continued downtrend in adherence (−15.0%) and mean adherence of 39.0% through W96.

**Table 2.** Week 96 Change in Virologic Status and Adherence

	Week 96 Virologic Suppression Status^a^			
	Virologic Suppression (HIV RNA <50 copies/mL)		Viremic (HIV RNA ≥50 copies/mL)	
No. (%)	19 (52.8)		17 (47.2)	
Mean PDC, W0–W96	78.0^b^		46.1^b^	
W48–W96 Change in Virologic Status	Continued Virologic Suppression	Achieved Virologic Suppression	Continued Viremia	Loss of Virologic Suppression
No. (%)	13 (36.1)	6 (16.7)	10 (27.8)	7 (19.4)
W96 HIV RNA, median (range), copies/mL	0 (0–49)	0 (0–0)	1315 (67–2 200 000)	93 (54–1190)
Mean PDC, W0–W96	81.0	71.0	39.0	59.0
Change in PDC (W48–W96 less W0–W48)	0.0	3.0	−15.0	−55.0

Abbreviations: B/F/TAF, bictegravir/emtricitabine/tenofovir alafenamide; PDC, percentage of days covered; W, week.

^a^All percentages were calculated based on the total number of participants in the observed, per-protocol analysis, n = 36.

^b^Significant differences observed. *P* < .01.

In subgroup analyses by adherence (PDC) level from W0 to W96, more participants achieved VS with adherence levels of ≥4 doses/wk (PDC ≥57%) (Figure 3*B*). This trend was consistently observed through W96. At W96, 16 participants with adherence of ≥57% achieved VS compared with 3 participants with adherence of <57% (*P* < .01).

### Retention in Care and Substance Use

Overall retention in care remained similar through W96, with 83.7% of participants retained in care through W96 (Supplementary Figure 3). Treatment-naïve participants (n = 12) had lower rates of visit retention at W96 compared with treatment-experienced participants (n = 31; 75.0% vs 87.1%; *P* = .34). Participants missed the same number of visits (mean missed visits, 3) during the W0–W48 and W48–W96 periods (mean difference in number of missed visits, 0.03; *P* = .95) (Supplementary Figure 2C).

At W96, 83.3% (n = 30/36) of participants had continued SUD. Methamphetamine remained the most common substance reported (80.6%). From W48 to W96, 2 participants (4.7%) ceased substance use, and 3 participants (7.0%) resumed SUD.

## DISCUSSION

The BASE study was the first study to evaluate the use of B/F/TAF among PWH/SUD. In the primary study period (W0–W48), we found B/F/TAF to be safe and effective despite suboptimal adherence, with 72% and 48% of participants achieving VS with HIV RNA <50 c/mL at W24 and W48, respectively (ITT) [20]. The present analysis retrospectively evaluated the BASE cohort 1 year after study completion (W48–W96). Compared with W0–W48, we found VS rates of 44% (ITT) with 18% lower B/F/TAF adherence during W48–W96. Despite lower B/F/TAF adherence rates, no evidence of treatment-emergent antiretroviral resistance was observed during W48–W96. Immunologic function increased through W96 but decreased during the W48–W96 period. Retention to care was stable through W96 (mean retention rate, 81%). SUDs, particularly with methamphetamines, remained prevalent (83%); however, a total of 6 participants (16%) had ceased substance use through W96. Our W96 results provide further support for B/F/TAF use as an unboosted, single-tablet regimen with a high barrier to antiretroviral resistance among populations at risk for incomplete adherence, such as PWH/SUD. Additionally, our results highlight the ongoing need for social and adherence support to encourage retention in HIV care and improve virologic outcomes among PWH/SUD.

SUD is associated with lower ART adherence [9, 10] and increased prevalence of HIV viremia [3, 5, 6] among PWH. Similar effects are evident in our retrospective W96 analysis when observing changes in virologic status from W48. We found that participants with continued HIV viremia or loss of VS at W96 exhibited lower adherence rates by −15% and −55%, respectively. Further, significantly lower adherence rates were observed in participants with HIV viremia compared with participants with VS (46.1% vs 78.0%) (Table 2). We hypothesize that the lower adherence rates in the retrospective analysis period (W48–W96) were potentially secondary to less patient interaction (eg, visit reminder calls, transportation assistance, etc.) and a lack of compensation for visit completion. Importantly, we observed no cases of treatment-emergent antiretroviral resistance from W48 to W96 despite the overall lower B/F/TAF adherence rates, further supporting B/F/TAF’s high barrier to antiretroviral adherence. Data from the ANRS 170 QUATUOR study showed that intermittent ART dosing (4 consecutive days on and 3 days off) was noninferior to daily dosing of ART [23]. Our results align with the ANRS 170 QUATUOR finding that participants taking ≥4 doses/wk were more likely to achieve VS. Recent data support the use of long-acting injectable cabotegravir/rilpivirine (CAB/RPV) in patients who are nonadherent to oral ART including patients with SUDs [24–26]. However, CAB/RPV may not be suitable for all patients, particularly populations prone to missing clinic visits [27], due to logistical considerations for retention in care for CAB/RPV (ie, patient tracking, bridging, etc.) [28]. Thus, an effective oral ART option with a high barrier to the development of antiretroviral resistance, such as B/F/TAF, is still needed. Specific adherence interventions should be considered for PWH/SUD to improve retention in care, virologic and overall health outcomes such as social support services (transportation, housing, etc.), efforts to reduce SUD-related stigma [29], and expanded SUD treatment (eg, opioid agonist therapy [OAT]) [3]. Further, there are very limited data assessing specific ART regimens in PWH/SUD populations [20, 26], and larger studies are needed to effectively assess the durability of modern ART regimens within this population.

B/F/TAF remained well tolerated during the W96 analysis. While nearly half of BASE participants experienced an AE through W96, none were attributed to B/F/TAF, led to study withdrawal, or caused a revision in ART away from B/F/TAF. While suicidal ideation was relatively common (n = 5, 11.6%) in the primary study period (W0–W48), there were fewer incidents of suicidal ideation during the retrospective study period (W48–W96), with 1 participant reporting 2 instances of suicidal ideation (5.6%). All instances of suicidal ideation were in conjunction with methamphetamine intoxication and deemed not related to B/F/TAF.

Interestingly, we found a mean weight and BMI change from W0 to W96 of 5.2 kg and 1.5 kg/m^2^, respectively. This could be associated with changes in lifestyle habits (eg, food selection, craving, and amounts) and/or treatment of mental health disorders (eg, antidepressant, antipsychotic, etc.) during tapering or cessation of substance use through W96. Additionally, these results are similar to other clinical trials showing that modern antiretrovirals, particularly a second-generation INSTI (BIC, DTG) + TAF, are associated with more weight gain than older antiretrovirals in ART-naïve PWH [30, 31]. Our results mirror the W96 analysis of the ADVANCE trial evaluating either DTG + F/TAF (TAF-arm) or DTG + F/tenofovir disoproxil fumurate (TDF-arm) vs the standard-of-care arm (SOC arm) of efavirenz + F/TDF in naïve PWH, with a mean weight change from W0 to W96 of 5.2 kg [31]. Real-world evidence from the BICSTaR study evaluating B/F/TAF use suggests potentially less weight gain (median [interquartile range]) in treatment-naïve (3.0 [0.5–8.0] kg) and -experienced patients (1.0 [−1.0 to 3.0] kg) [32]. We found a small, nonsignificant decrease in mean weight gain from W48 to W96 compared with W0 to W48 (W0–W48: 2.8 kg; vs W48–W96: 2.3 kg). While this may suggest a slight tapering effect of weight gain with time, the overall trend is generally upward through W96. Further, associations of other cardiometabolic implications (eg, hypertension, diabetes mellitus, nonalcoholic fatty liver disease) have been observed with INSTI-based ART [31, 33, 34]. In addition to social support, adherence, and SUD treatment services, clinicians should consider additional interventions focused on cardiovascular health to encourage healthy lifestyle habits, particularly dietary habits, as food insecurity is commonly associated with PWH/SUD [35].

During W48–W96, we observed participant transitions in retention in care (Supplementary Figure 1), with 7 participants who did not return to care by W48 and 2 participants who met W48 but were lost to follow-up through W96. Yet, our overall retention in care rate was stable through W96, with a mean visit retention of 81% across all study time points. These results are encouraging considering that a recent 2-year study observed lower rates of retention in care in PWH/SUD (67%) and PWH (76%) [36]; our results may be a result of trust-building developed through BASE study participation. Further, we observed no significant difference in missed HIV care visits between W0–W48 and W48–W96 (Supplementary Figure 2C), even though participants returned to clinical care and did not receive further financial incentives ($20 Visa cash card) after W48. Engagement in HIV care is highly intertwined with removing SUD-related stigma, providing a welcoming, nonjudgmental clinic experience, and fostering transparent communication to develop patient–provider trust (Heerten-Rodriquez et al., unpublished data). These care delivery aspects may help to encourage SUD de-escalation and/or cessation through OAT intervention and/or SUD treatment. SUD cessation was similar between study periods (W0–W48: 11.6% [n = 5/43]; W48–W96: 14.0% [n = 6/43]). All participants achieving SUD cessation received care through referrals to an intensive outpatient treatment program focused on combined mental health and SUD treatment. All participants achieving SUD cessation had VS at W96. Participants with ongoing SUD at W96 missed more HIV care visits (median [range] missed visits, 3 [1–9]) compared with participants ceasing SUD (median [range] missed visits, 2 [0–3]), further highlighting the association of SUD with retention in care and health outcomes.

Our study has limitations. Most notably, the generalizability of our results is limited by our small sample size from a single cohort. Further, the W96 analysis was retrospective and could have led to information bias. Larger studies are needed to validate our findings.

During W48–W96 of the retrospective analysis of the BASE cohort, the proportion of PWH/SUD achieving VS with B/F/TAF decreased to 44%, along with a subsequent decrease in adherence by −18%. Despite lower B/F/TAF adherence, no emergent antiretroviral resistance occurred, and retention in care was stable through W96. Our data complement the existing literature on B/F/TAF, supporting its use among PWH/SUD, and generally reinforce its high barrier to the development of antiretroviral resistance.

## Supplementary Material

ofae737_Supplementary_Data

## Supplementary Data

Supplementary materials are available at *Open Forum Infectious Diseases* online. Consisting of data provided by the authors to benefit the reader, the posted materials are not copyedited and are the sole responsibility of the authors, so questions or comments should be addressed to the corresponding author.
