# Supplementary material for: Week 96 Results of Bictegravir/Emtricitabine/Tenofovir Alafenamide for HIV Treatment in People With Substance Use Disorders
Source: Open Forum Infect Dis. 2024 Dec 20;12(1):ofae737. doi: 10.1093/ofid/ofae737 (PMC11713015; doi:10.1093/ofid/ofae737)
Supplement: ofae737_Supplementary_Data [file ofae737_supplementary_data.zip › BASE96 Supplementary Table.docx]

| Participant Number | Week 24,  HIV-1 RNA (c/mL) | Week 48,  HIV-1 RNA (c/mL) | Week 48  Disposition | Week 96,  HIV-1 RNA (c/mL) | Genotyping | Week 96  Disposition | Week 96  ART |
| --- | --- | --- | --- | --- | --- | --- | --- |
| 12 | <20 | 69500 | PDVF; LTF | 144000 | No | LTF | B/F/TAF |
| 15 | 59 | <20 | Suppressed | 1190 | No^a^ | LTF | B/F/TAF |
| 20 | 1410 | 77 | LTF | 690 | Yes, Pansensitive | Reengaged in care | B/F/TAF |
| 32 | 452000 | ND | LTF | 2200000 | Yes, Pansensitive | In care | B/F/TAF |
| 35 | ND | ND | LTF | 1940 | No^b^ | Reengaged in care | DTG + F/TAF |
| 37 | ND | ND | LTF | 1730000 | No^b^ | Reengaged in care | B/F/TAF |
| 39 | 18000 | 9620 | PDVF; LTF | 307000 | No^b^ | Reengaged in care | B/F/TAF |

Supplementary Table 1. Characteristics of participants meeting protocol-defined virologic failure at week 96.

^a^Participant deceased shortly after week 96.

^b^Participants were off ART for >3 months upon return to care.

Definitions: c/mL, copies/milliliter; PDVF, protocol-defined virologic failure; LTF, lost to follow-up; B/F/TAF, bictegravir/emtricitabine/tenofovir alafenamide; DTG, dolutegravir.
