# Supplementary figures and images for: Week 96 Results of Bictegravir/Emtricitabine/Tenofovir Alafenamide for HIV Treatment in People With Substance Use Disorders
Source: Open Forum Infect Dis. 2024 Dec 20;12(1):ofae737. doi: 10.1093/ofid/ofae737 (PMC11713015; doi:10.1093/ofid/ofae737)

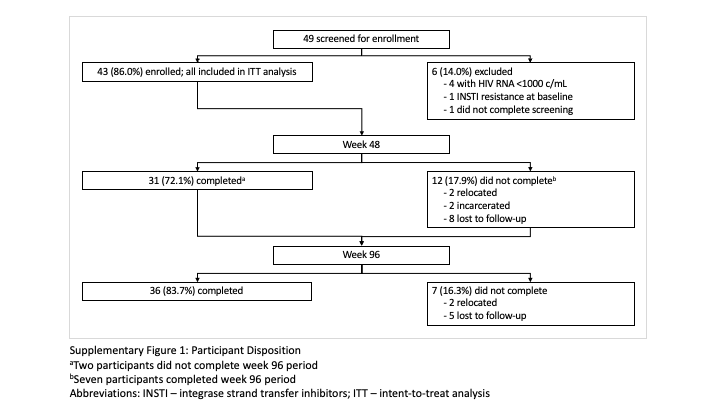

Supplement: ofae737_Supplementary_Data [file ofae737_supplementary_data.zip › SupplementaryFig1_W96.Disposition.tiff]

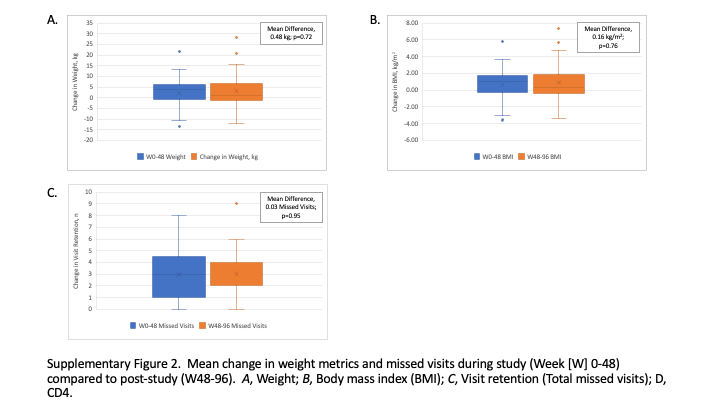

Supplement: ofae737_Supplementary_Data [file ofae737_supplementary_data.zip › SupplementaryFig2.Wk96.Change_Wt.BMI.Retention.tiff]

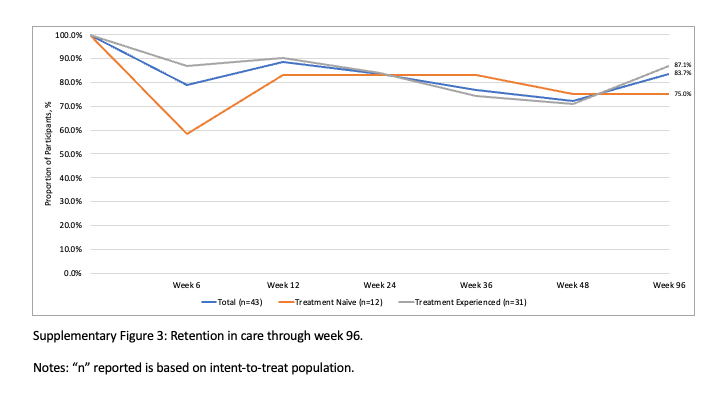

Supplement: ofae737_Supplementary_Data [file ofae737_supplementary_data.zip › SupplementaryFig3.Retention.tiff]
